# Quality Assessment of Urinary Stone Analysis: Results of a Multicenter Study of Laboratories in Europe

**DOI:** 10.1371/journal.pone.0156606

**Published:** 2016-06-01

**Authors:** Roswitha Siener, Noor Buchholz, Michel Daudon, Bernhard Hess, Thomas Knoll, Palle J. Osther, José Reis-Santos, Kemal Sarica, Olivier Traxer, Alberto Trinchieri, EAU Section of Urolithiasis (EULIS)

**Affiliations:** 1 University Stone Centre, Department of Urology, University of Bonn, Bonn, Germany; 2 Endourology and Stone Services, Bart's and the London NHS Trust, London, United Kingdom; 3 APHP, Service des Explorations Fonctionnelles, Tenon University Hospital, Pierre and Marie Curie University, Paris, France; 4 Internal Medicine and Nephrology, Klinik im Park and University of Zürich, Zürich, Switzerland; 5 Department of Urology, Klinikum Sindelfingen-Böblingen, University of Tübingen, Sindelfingen, Germany; 6 Urological Research Center, Department of Urology, Lillebaelt Hospital, University of Southern Denmark, Fredericia, Denmark; 7 Faculdade de Engenharia Clínica e Biomédica da Universidade Católica Portuguesa, Lisbon, Portugal; 8 Department of Urology, Dr Lufti Kirdar Kartal Research and Training Hospital, Istanbul, Turkey; 9 Department of Urology, Tenon University Hospital, Pierre and Marie Curie University, Paris, France; 10 Department of Urology, A. Manzoni Hospital, Lecco, Italy; IPK, GERMANY

## Abstract

After stone removal, accurate analysis of urinary stone composition is the most crucial laboratory diagnostic procedure for the treatment and recurrence prevention in the stone-forming patient. The most common techniques for routine analysis of stones are infrared spectroscopy, X-ray diffraction and chemical analysis. The aim of the present study was to assess the quality of urinary stone analysis of laboratories in Europe. Nine laboratories from eight European countries participated in six quality control surveys for urinary calculi analyses of the Reference Institute for Bioanalytics, Bonn, Germany, between 2010 and 2014. Each participant received the same blinded test samples for stone analysis. A total of 24 samples, comprising pure substances and mixtures of two or three components, were analysed. The evaluation of the quality of the laboratory in the present study was based on the attainment of 75% of the maximum total points, i.e. 99 points. The methods of stone analysis used were infrared spectroscopy (n = 7), chemical analysis (n = 1) and X-ray diffraction (n = 1). In the present study only 56% of the laboratories, four using infrared spectroscopy and one using X-ray diffraction, fulfilled the quality requirements. According to the current standard, chemical analysis is considered to be insufficient for stone analysis, whereas infrared spectroscopy or X-ray diffraction is mandatory. However, the poor results of infrared spectroscopy highlight the importance of equipment, reference spectra and qualification of the staff for an accurate analysis of stone composition. Regular quality control is essential in carrying out routine stone analysis.

## Introduction

Prevalence and incidence of urolithiasis in industrialized countries have markedly increased over the past decades. The prevalence of urinary stone disease in the Unites States significantly increased from 5.2% in 1988 to 1994 to 8.8% in 2007 to 2010 [1,2]. In Japan, a rise in the prevalence from 4.0% to 5.4% was observed within 10 years [3]. In Germany, the prevalence of urolithiasis markedly increased from 4.0% to 4.7% and the incidence from 0.54% to 1.47% between 1979 and 2001 [4].

The recurrence rate of urinary stones is estimated to be up to 42% [4,5,6]. The high incidence of recurrence indicates that metaphylactic measures after stone removal are still inadequate. Patients at high risk of recurrent stone formation are those with infection stones, uric acid, urate (i.e. monoammonium urate, monopotassium urate and monosodium urate monohydrate), brushite and genetically determined stones (i.e. cystine, 2,8-dihydroxyadenine and xanthine stones) [7]. Depending on different risk factors, calcium oxalate stone disease is likewise characterised by a high frequency of recurrence [8]. For effective management of the stone-forming patient, accurate stone analysis is, therefore, an essential component of the diagnostic work-up and a prerequisite of metabolic evaluation [9].

According to the EAU guidelines (2015), all patients should have at least one stone analysed [7]. As the stone composition has significant therapeutic importance in the evaluation of patients, all urinary stones should be analysed [10]. Different techniques have been used for the compositional analysis of urinary stones, including X-ray diffraction (XD), infrared spectroscopy (IR) and chemical analysis (CA). Whereas IR is used for the examination of chemical molecular structures, XD is used for the determination of the crystalline structure of a substance. For the correct analysis of stone composition, IR and XD offer the highest degree of certainty. Due to poor results, wet chemical analysis of urinary stones is considered to be obsolete [10].

Urinary stones are often composed of more than one substance, which presents a difficulty in accurate assessment of the stone composition. A study performed in the United States found that commercial laboratories reliably recognised pure calculi, whereas variability in the reporting of mixtures was observed [11]. Analysis of more than 10.000 human urinary calculi revealed that only 7% of stones consisted of just one component [12], reflecting the clinical relevance of such a test variability.

Data regarding the accuracy of urinary stone analysis in Europe is lacking, stressing the importance of quality assessment of stone analysis in Europe. The application of analytical methods for stone analysis and the quality of the results were evaluated in nine European stone analysis laboratories participating in quality control surveys for urinary calculi analyses.

## Materials and Methods

Nine urinary stone analysis laboratories from eight European countries (Denmark, France, Germany, Italy, Portugal, Switzerland, Turkey and United Kingdom) participated in six quality control surveys for urinary calculi analyses by the Reference Institute for Bioanalytics (RfB), Bonn, Germany, between 2010 and 2014. Of the nine laboratories, four had already participated in previous quality control surveys conducted by the RfB. Each participant received the same 24 blinded test samples for stone analysis to allow for direct comparison.

All laboratories advertised the use of their method for stone analysis. The certification of the analytical results by the RfB was based primarily on the correct qualitative proof of the substances present in each sample. A correct result required precise qualitative determination in mixtures at 10% gradations. For evaluation, the following scoring system was used:

**Table:** 

Substance present ≥ 80%	+4 points
Substance present between 30 and 70%	+3 points
Substance present ≤ 20%	+2 points
per false analysis (but the score for one sample cannot become negative)	-1 point

Simplified analysis results (e.g. calcium oxalate instead of calcium oxalate monohydrate or calcium oxalate dihydrate; calcium phosphate instead of carbonate apatite, apatite, brushite or whitlockite; uric acid instead of uric acid dihydrate) scored with half of the possible points.

In order to get the certificate for each survey, at least 75% of the maximum points were needed. If the analysis of the laboratory yielded only simplified analyses results (e.g. calcium oxalate instead of calcium oxalate monohydrate or calcium oxalate dihydrate), then the laboratory would normally fail in achieving the required number of points. A total of 132 points could be maximally achieved by each laboratory after participation in the present six surveys. The evaluation of the quality of a laboratory in the present study was based on the attainment of 75% of the maximum total points, i.e. 99 points.

The samples used as test substances, single components and mixtures are shown in Table 1. The mixtures comprised two or three components of varying percentages by weight. While the majority of the samples were synthetic, the reference material for the remaining samples consisted of native urinary calculi. The use of synthetic products as test substances is necessary, as sufficient amounts of native urinary stone material are rarely available in the high quantities needed for the large number of participants in these surveys. The advantage of synthetic material is that these substances are standardised for purity and crystallinity, whereas urinary stones frequently contain mixtures and differ strongly in their crystallinity [13]. The test substances for the quality control surveys for urinary calculi analyses of the RfB were obtained from Dr. G. Schubert, Vivantes Klinikum im Friedrichshain, Institute of Laboratory Diagnostics, Berlin, Germany.

**Table 1:** Test substances for analysis.

Sample	Proportion	Chemical name	Mineralogical name	Reference material
		**Pure**		
**1**	100%	Calcium carbonate (n = 2)	Calcite	Fluka
**2**	100%	Calcium phosphate	Whitlockite	Fluka
**3**	100%	Cholesterol (n = 2)	-	Aldrich
**4**	100%	Monopotassium urate	-	Sigma
**5**	100%	Monosodium urate monohydrate	-	Sigma
**6**	100%	Sucrose	Cristal sugar	Cristal sugar
		**Two components**		
**7**	70%	Calcium hydrogen phosphate dihydrate	Brushite	Fluka
	30%	Calcium oxalate monohydrate	Whewellite	
**8**	50%	Calcium hydrogen phosphate dihydrate	Brushite	Fluka
	50%	Calcium oxalate monohydrate	Whewellite	
**9**	60%	Calcium hydrogen phosphate dihydrate	Brushite	Fluka
	40%	Apatite	Apatite	
**10**	70%	Calcium oxalate monohydrate	Whewellite	Native urinary calculus
	30%	Apatite	Apatite	
**11**	60%	Cystine	-	Fluka
	40%	Calcium oxalate monohydrate	Whewellite	
**12**	20%	Cystine	-	Fluka
	80%	Calcium oxalate monohydrate	Whewellite	
**13**	70%	Magnesium ammonium phosphate hexahydrate	Struvite	Native urinary calculus
	30%	Apatite (n = 2)	Apatite	
**14**	30%	Magnesium ammonium phosphate hexahydrate	Struvite	Native urinary calculus
	70%	Apatite	Apatite	
**15**	60%	Uric acid dihydrate	-	Native urinary calculus
	40%	Uric acid	Uricite	
**16**	30%	Uric acid dihydrate	-	Native urinary calculus
	70%	Uric acid	Uricite	
**17**	80%	Uric acid	Uricite	Native urinary calculus
	20%	Calcium oxalate monohydrate	Whewellite	
**18**	70%	Uric acid	Uricite	Fluka
	30%	Calcium oxalate monohydrate	Whewellite	
**19**	50%	Uric acid	Uricite	Native urinary calculus
	50%	Calcium oxalate monohydrate	Whewellite	
		**Three components**		
**20**	50%	Calcium oxalate dihydrate	Weddellite	Native urinary calculus
	30%	Calcium oxalate monohydrate	Whewellite	
	20%	Apatite	Apatite	
**21**	50%	Magnesium ammonium phosphate hexahydrate	Struvite	Native urinary calculus
	30%	Apatite	Apatite	
	20%	Calcium oxalate monohydrate	Whewellite	

## Results

The method of analysis most frequently used by participating laboratories was IR (n = 7). One laboratory reported the application of XD and another of CA. The comparison of the analytical results of the nine laboratories and the correct composition of the samples for stone analysis are presented in Table 2. The error rates of qualitative analysis for test substances of single components and components in mixtures are shown in Table 3.

**Table 2:** Comparison of analytical results of nine laboratories and the correct composition of samples for stone analysis.

			Lab A	Lab B	Lab C	Lab D	Lab E	Lab F	Lab G	Lab H	Lab I
			XD	IR	IR	IR	IR	IR	IR	IR	CD
Sample	Substances	%	%	%	%	%	%	%	%	%	%
**Survey 1**											
**A**	Cholesterol	100	100	100	100	100	100	100	0	0	0
	False analysis	-	-	-	-	-	-	-	100 MP	100 AF	100 NI
**B**	Uric acid dihydrate	60	50	50	0	60	30	0	0	0	0
	Uricite	40	50	50	80	40	60	0	0	100	100
	False analysis	-	-	-	20 WD	-	10 WH	100 UA	100 UA	-	-
**C**	Brushite	50	60	70	50	60	30	50	100	50	0
	Whewellite	50	40	30	50	40	50	0	0	0	40
	False analysis	-	-	-	-	-	20 WD	50 CaOx	-	50 WD	30 WD
	False analysis	-	-	-	-	-	-	-	-	-	30 AP
**D**	Monopotassium urate	100	100	100	100	100	100	0	0	0	0
	False analysis	-	-	-	-	-	-	100 UA	100 MU	100 MU	100 NI
**Survey 2**											
**A**	Calcite	100	100	100	100	100	100	100	100	0	0
	False analysis	-	-	-	-	-	-	-	-	100 NI	100 NI
**B**	Uricite	50	30	60	50	70	35	0	100	50	0
	Whewellite	50	70	30	50	30	65	0	0	50	0
	False analysis	-	-	10 UD	-	-	-	65 CaOx	-	-	100 NI
	False analysis	-	-	-	-	-	-	35 UA	-	-	-
**C**	Cystine	60	80	60	60	60	55	70	100	90	100
	Whewellite	40	20	40	40	40	45	0	0	10	0
	False analysis	-	-	-	-	-	-	30 CaOx	-	-	-
**D**	Whitlockite	100	90	100	0	0	0	0	0	0	0
	False analysis	-	10 NI	-	100 NI	100 NI	100 AF	100 CP	100 NI	100 NI	100 NI
**Survey 3**											
**A**	Whewellite	70	70	30	40	40	55	0	25	60	0
	Apatite	30	30	70	60	60	40	65	75	40	0
	False analysis	-	-	-	-	-	5 PR	35 CaOx	-	-	100 NI
**B**	Struvite	70	80	80	60	80	50	0	35	40	0
	Apatite	30	20	20	40	20	40	25	65	60	0
	False analysis	-	-	-	-	-	10 PR	75 MP	-	-	100 NI
**C**	Weddellite	50	40	40	40	40	65	0	0	85	0
	Whewellite	30	40	30	30	40	20	0	50	0	0
	Apatite	20	20	30	30	20	15	10	50	15	0
	False analysis	-	-	-	-	-	-	90 CaOx	-	-	100 NI
**D**	Struvite	50	20	20	30	60	20	0	100	10	0
	Apatite	30	70	70	70	30	70	45	0	75	0
	Whewellite	20	10	10	0	0	10	0	0	10	0
	False analysis	-	-	-	-	10 WD	-	55 MP	-	5 WD	100 NI
**Survey 4**											
**A**	Whewellite	80	70	60	80	70	90	0	0	100	0
	Cystine	20	30	40	20	30	10	50	95	0	100
	False analysis	-	-	-	-	-	-	50 CaOx	5 BR	-	-
**B**	Cholesterol	100	100	100	100	100	100	100	0	0	0
	False analysis	-	-	-	-	-	-	-	100 CY	100 NI	100 NI
**C**	Monosodium urate monohydrate	100	100	100	100	100	100	100	100	100	0
	False analysis	-	-	-	-	-	-	-	-	-	100 NI
**D**	Brushite	70	80	80	80	70	65	80	100	0	0
	Whewellite	30	20	20	20	30	35	0	0	0	0
	False analysis	-	-	-	-	-	-	20 CaOx	-	100 WL	100 NI
**Survey 5**											
**A**	Apatite	70	60	30	50	40	80	60	100	80	0
	Struvite	30	30	70	40	60	15	0	0	15	0
	False analysis	-	10 WH	-	10 WH	-	5 WH	40 MP	-	5 PR	100 NI
**B**	Uricite	70	50	80	60	50	60	0	0	85	0
	Uric acid dihydrate	30	50	20	40	50	40	0	100	15	0
	False analysis	-	-	-	-	-	-	100 UA	-	-	100 NI
**C**	Calcite	100	100	100	100	100	100	100	100	0	0
	False analysis	-	-	-	-	-	-	-	-	100 NI	100 NI
**D**	Uricite	80	20	50	40	40	50	0	100	50	0
	Whewellite	20	80	50	60	60	50	0	0	50	0
	False analysis	-	-	-	-	-	-	55 UA	-	-	100 NI
	False analysis	-	-	-	-	-	-	45 CaOx	-	-	-
**Survey 6**											
**A**	Uricite	70	70	60	70	60	60	0	0	80	0
	Whewellite	30	30	40	30	40	40	0	0	20	0
	False analysis	-	-	-	-	-	-	45 CaOx	100 NI	-	100 NI
	False analysis	-	-	-	-	-	-	55 UA	-	-	-
**B**	Struvite	70	50	70	70	70	55	0	35	40	0
	Apatite	30	50	30	30	30	40	30	0	60	0
	False analysis	-	-	-	-	-	5 PR	70 MP	65 WL	-	100 NI
**C**	Brushite	60	80	60	50	30	45	80	0	0	0
	Apatite	40	20	40	50	70	55	20	65	30	0
	False analysis	-	-	-	-	-	-	-	35 ST	70 WL	100 NI
**D**	Sucrose	100	100	100	100	100	100	100	0	0	0
	False analysis	-	-	-	-	-	-	-	100 NI	100 NI	100 NI

AF: artifact; AP: apatite; BR: brushite; CaOx: calcium oxalate (simplified analysis result); CP: calcium phosphate (simplified analysis result); CY: cystine; MP: magnesium ammonium phosphate (simplified analysis result); MU: monoammonium urate; NI: non-identifiable; PR: protein; ST: struvite; UA: uric acid (simplified analysis result); UD: uric acid dihydrate; WD: weddellite; WH: whewellite; WL: whitlockite

**Table 3:** Error rates of qualitative analysis for test substances.

Substances	Incorrect qualitative analysis (%)			Total
	IR (n = 7)	CA (n = 1)	XD (n = 1)	
**Substances in mixtures**				
Calcium oxalate monohydrate (whewellite)	32.9% (23/70)	90% (9/10)	0% (0/10)	35.6% (32/90)
Calcium oxalate dihydrate (weddellite)	28.6% (2/7)	100% (1/1)	0% (0/1)	33.3% (3/9)
Apatite	4.1% (2/49)	100% (7/7)	0% (0/7)	14.3% (9/63)
Calcium hydrogen phosphate dihydrate (brushite)	14.3% (3/21)	100% (3/3)	0% (0/3)	22.2% (6/27)
Magnesium ammonium phosphate hexahydrate (struvite)	17.9% (5/28)	100% (4/4)	0% (0/4)	25% (9/36)
Uric acid	22.9% (8/35)	80% (4/5)	0% (0/5)	26.7% (12/45)
Uric acid dihydrate	35.7% (5/14)	100% (2/2)	0% (0/2)	38.9% (7/18)
Cystine	7.1% (1/14)	0% (0/2)	0% (0/2)	6.3% (1/16)
**Pure substances**				
Calcium carbonate (calcite)	14.3% (2/14)	100% (2/2)	0% (0/2)	22.2% (4/18)
Calcium phosphate (whitlockite)	85.7% (6/7)	100% (1/1)	0% (0/1)	77.8% (7/9)
Cholesterol	28.6 (4/14)	100% (2/2)	0% (0/2)	33.3% (6/18)
Monopotassium urate	42.9 (3/7)	100% (1/1)	0% (0/1)	44.4% (4/9)
Monosodium urate monohydrate	0% (0/7)	100% (1/1)	0% (0/1)	11.1% (1/9)
Sucrose	28.6% (2/7)	100% (1/1)	0% (0/1)	33.3% (3/9)

The error rate of up to 100% was extremely high for the laboratory using CA. Regarding the most common stone components (i.e. whewellite, uric acid and apatite), the error rate for CA was between 80 and 100%. IR showed incorrect results in up to 86% of the analyses. Although XD provided correct detection of each substance, the laboratory did not receive the maximum total points of 132 due to 2 false-positive analyses of substances.

Whewellite, one of the most common stone components, was either not found or wrongly identified in 36% (32 of 90) of analyses. The error rates were 33% (23 of 70) for IR, 90% for CA and 0% for XD.

Seventy-three percent of all participants reliably recognised uric acid. However, only 61% (11 of 18) of analyses correctly identified the component uric acid dihydrate. The error rates in the case of uric acid and uric acid dihydrate when using IR were 23% and 36%, respectively. The laboratory using CA did not identify uric acid dihydrate, whereas the laboratory with XD correctly reported uric acid and uric acid dihydrate.

Brushite was reliably recognized in nearly 80% and apatite in 86% of analyses. Whereas the laboratory using CA was unable to identify brushite and apatite, XD correctly provided detection of both substances in mixtures. Error rates regarding analyses of brushite and apatite were 14% and 4%, respectively, for IR.

The single components comprised the biliary calculus substance cholesterol, the rare stone components whitlockite, monopotassium urate, monosodium urate monohydrate and calcium carbonate and the artifact sucrose, which occasionally occurs in stone analysis laboratories. Error rates of up to nearly 80% of all analyses were recorded. Whereas the laboratory using XD correctly determined each of these substances, the participant using CA was unable to identify any of these components. The error rates for IR were between 0% and 86%, depending on the substance.

**Fig 1:**
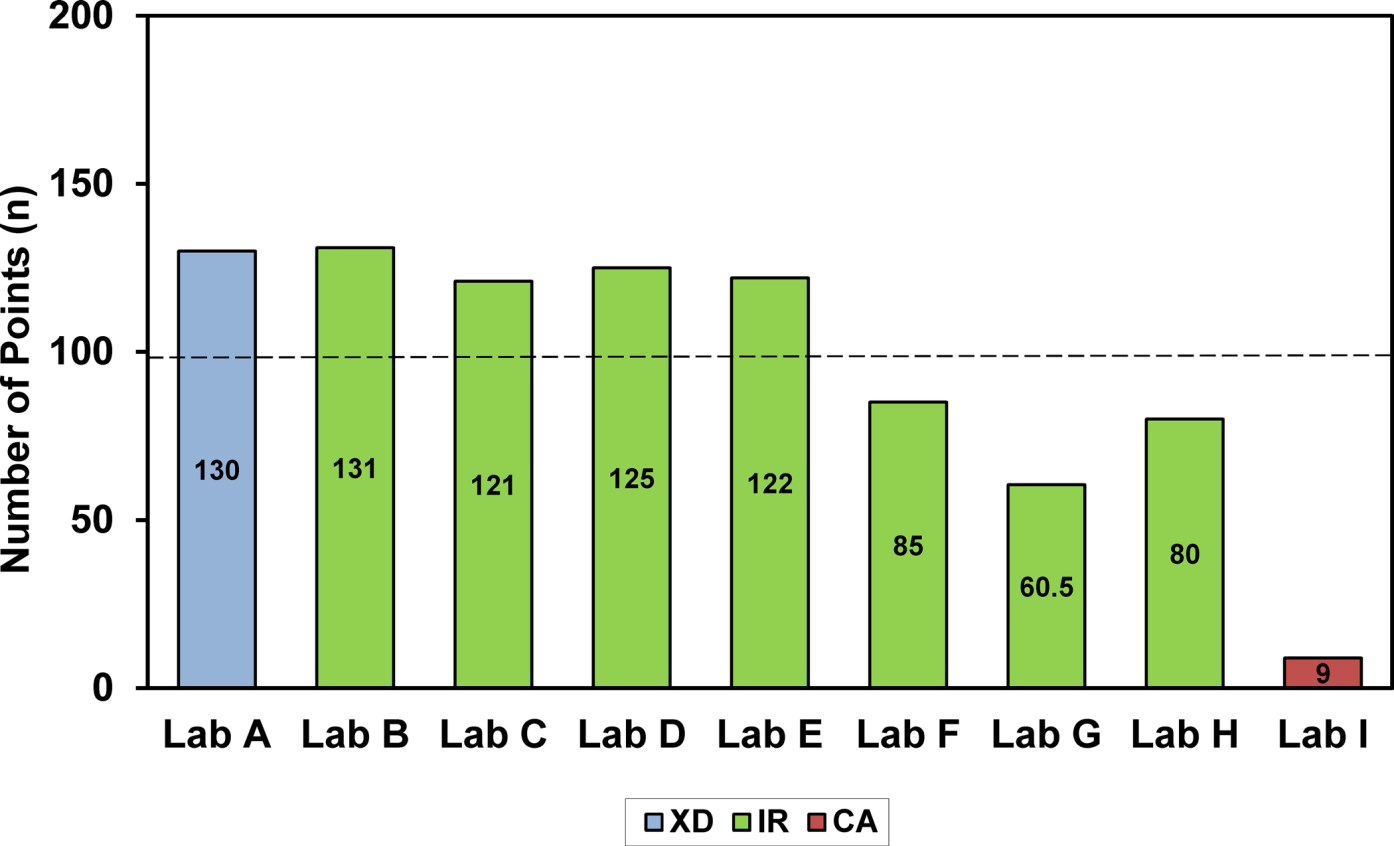
Evaluation of analytical results of laboratories (maximum: 132 points; minimum: 99 points).

In the current study, only 56% (5 of 9) of the participants fulfilled the quality requirements of at least 99 points (Fig 1).

## Discussion

Appropriate and thorough analysis of urinary stone composition is the most crucial laboratory diagnostic procedure and the basis for dietary and medical treatment of the stone-forming patient [7,9,14]. Compositional stone analysis should, therefore, be an integral part of the metabolic evaluation of patients with urolithiasis [15]. Incorrect analysis or failure to identify a stone substance may result in inadequate therapy.

Two previous studies based on the analysis of more than 50,000 and 200,000 urinary stones, respectively, revealed a significant increase in the occurrence of brushite stones during recent years [16,17]. Brushite stones are known to grow rapidly with a correspondingly very high recurrence rate. Effective treatment is therefore highly important in brushite stone disease [18]. Additionally, the composition of a stone can be an important factor in its fragility. Due to their hardness, brushite and whewellite (calcium oxalate monohydrate) have been shown to respond poorly to disintegration by Extracorporeal Shock Wave Lithotripsy (ESWL) [7,19]. Whewellite is among the most common urinary stone substances. The correct qualitative determination and differentiation of whewellite and brushite is therefore essential for a selective approach to stone therapy. The participant using CA was unable to identify whewellite in 90% of the analyses, but incorrect results were also found in one third of analyses (33%) using IR. While the laboratory with CA could not detect brushite, IR showed incorrect results in 14% of the analyses. The participant with XD correctly reported brushite and whewellite in each sample.

Compared to brushite, the mixture of uric acid and uric acid dihydrate is relatively common. A recent study based on the analysis of 43,545 stones found that uric acid stone composition increased markedly in both sexes above the age of 50 years [20]. The differentiation between uric acid and uric acid dihydrate can provide valuable information regarding the pathogenesis of the stone, because uric acid dihydrate is formed in very acidic urine [9]. While more than 70% of all analyses reliably recorded uric acid, only 61% of laboratories correctly detected the component uric acid dihydrate. CA was unable to identify uric acid dihydrate; however, 5 of 14 (36%) of analyses using IR also failed.

Among the pure substances, only monosodium urate monohydrate, a rare urinary stone component, was correctly determined by IR. On the contrary, laboratories using IR were unable to identify whitlockite (calcium phosphate) in 86% of the analyses. Whereas XD showed correct analyses of all pure substances, the error rate of CA was 100%.

It is not only undetected urinary stone components that may lead to inaccurate diagnosis and subsequent inadequate recurrence prevention, but also false-positive analysis of substances that are not present in the stone. For instance, incorrect analysis specifying the urinary calculus substance cystine or magnesium ammonium phosphate instead of cholesterol is more detrimental to the patient than the statement “not identifiable”.

Several studies revealed that CA exhibits very high error rates of up to 94%, depending on the stone component [10,21,22]. Our study in nine European laboratories confirms the high error rates with the application of CA in the analysis of pure substances as well as mixtures. Regarding the most common stone components (i.e. whewellite, uric acid and apatite), the error rate for CA was between 80 and 100%. According to the current standard, CA is considered to be insufficient for stone analysis, whereas IR or XD is mandatory [7,10]. Among the different methods for urinary stone analysis, chemical analysis has been traditionally used most widely due to its low cost. While XD requires more expensive equipment than IR, the advantage of IR is the moderate cost.

Surprisingly, the evaluation of the results of our study revealed high error rates for both pure substances and mixtures also in laboratories using IR, which is in contrast to previous findings [10,21,22]. Of the 7 laboratories that used IR, just 4 (57%) fulfilled the quality requirements. Except for uric acid dihydrate, which is difficult to identify by IR in a mixture with uric acid, all other pure substances and mixtures would have been expected to be correctly detected. The results of our study showed that high performance can also be achieved by IR, but it requires the use of sophisticated equipment and qualified personnel. Most computer programs for the automated evaluation of IR spectra and XD diagrams are considered to provide unreliable component identification of mixtures [23]. The quality of the library of reference spectra is regarded as the major factor contributing to the reliability of the evaluation software [13]. Evaluation of the analysis carried out using IR and XD should always be checked by qualified personnel [10]. The results underline previous findings that the equipment, reference spectra and trained staff are indispensable preconditions for an accurate application of IR [10,13].

## Conclusions

Targeted recurrence prevention requires reliable information on stone composition. For laboratories that are not able to offer sophisticated analysis techniques and interpretative expertise, referral or centralisation of stone analysis seems to be the only recommendable approach.

In order to ensure reliable results, measures for quality control of urinary stone analysis are required. Among the five laboratories in Europe that fulfilled the quality requirements, four of these laboratories had already participated in previous quality control surveys for urinary calculi analyses. It can be concluded that regular quality control is essential in carrying out routine stone analysis
